# *Synemaguiyang* sp. nov., the fourth endemic species of *Synema* Simon, 1864 (Araneae, Thomisidae) from China

**DOI:** 10.3897/BDJ.10.e85072

**Published:** 2022-05-12

**Authors:** Jianshuang Zhang, Wanling Zhang, Langju Deng, Qianle Lu, Hao Yu

**Affiliations:** 1 The State Key Laboratory of Southwest Karst Mountain Biodiversity Conservation of Forestry Administration, School of life sciences, Guizhou Normal University, Guiyang, China The State Key Laboratory of Southwest Karst Mountain Biodiversity Conservation of Forestry Administration, School of life sciences, Guizhou Normal University Guiyang China; 2 The Key Laboratory of Plant Physiology and Development in Guizhou Province, School of life sciences, Guizhou Normal University, Guiyang, China The Key Laboratory of Plant Physiology and Development in Guizhou Province, School of life sciences, Guizhou Normal University Guiyang China; 3 School of Biological Sciences, Guizhou Education University, Guiyang, China School of Biological Sciences, Guizhou Education University Guiyang China; 4 College of Life Sciences and Oceanography, Shenzhen University, Shenzhen, China College of Life Sciences and Oceanography, Shenzhen University Shenzhen China

**Keywords:** crab spiders, morphology, DNA barcoding, diagnosis, taxonomy

## Abstract

**Background:**

*Synema* Simon, 1864 is a relatively large genus of family Thomisidae Sundevall, 1833 and currently includes 124 species distributed worldwide, except for the Polar Regions. However, *Synema* can be regarded as being poorly represented in China, with only seven species, three of which are endemic.

**New information:**

A new spider species of the genus *Synema* from Guiyang City in China, is described under the name of *S.guiyang* J. Zhang, Q. Lu & H. Yu, sp. nov. Detailed descriptions and photographs of the new species are provided. DNA barcodes (a partial fragment of the mitochondrial cytochrome oxidase subunit I gene, COI) of the species were obtained to confirm matching of the sexes and for future use in molecular studies.

## Introduction

The Thomisidae is one of the largest spider families, with 171 genera and 2159 valid species distributed worldwide, 51 genera and 306 species of which are recorded from China (World Spider Catalog 2022). Most of pioneer studies on the crab spider of China were carried out by Mingsheng Zhu, and concentrated presented in an indispensable publication: *Fauna Sinica: Arachnida: Araneae: Thomisidae* (Song and Zhu 1997, in Chinese). In the past nearly 20 years, the Chinese Thomisids have been well-studied by Dr. Guo Tang, Dr. Keke Liu, Mr. Yejie Lin and their co-authors etc., along with high quality illustrations and descriptions (Tang et al. 2005, Tang et al. 2006, Tang et al. 2007, Tang et al. 2008a, Tang et al. 2008b, Tang et al. 2008c, Tang and Li 2009a, Tang and Li 2009b, Tang and Li 2009c, Tang et al. 2009, Tang and Li 2010a, Tang and Li 2010b, Tang et al. 2010, Tang and Li 2012, Tang et al. 2013, Liu et al. 2017, Lin et al. 2019, Meng et al. 2019, Lin et al. 2022, Liu et al. 2022). However, there are still many species requiring study (Liu et al. 2022).

*Synema* is the fourth most speciose genus of Thomisidae and includes 124 species so far, after *Xysticus* C. L. Koch, 1835 (293 species), *Tmarus* Simon, 1875 (226 species) and *Thomisus* Walckenaer, 1805 (143 species) (*World Spider Catalog 2022*). Currently, only seven *Synema* species are known from China, amongst them, three are endemic (*World Spider Catalog 2022*). Despite being common, the genus remains inadequately studied because: more than half of the species are known from a single sex or juveniles (34 from males, 39 from females, 8 from juveniles) (*World Spider Catalog 2022*); original descriptions are rather brief and lack illustrations or illustrations are inadequate (Tang and Li 2010b); types of some species do not exist or are difficult to locate or access (World Spider Catalog 2022); the diversity of this genus is still insufficiently known. However, in spite of the dispute about the limit of genus, most of the *Synema* species from the Oriental realm have been well-described in detail, alongside high quality illustrations, to allow for easy species recognition.

**Figure 1.**
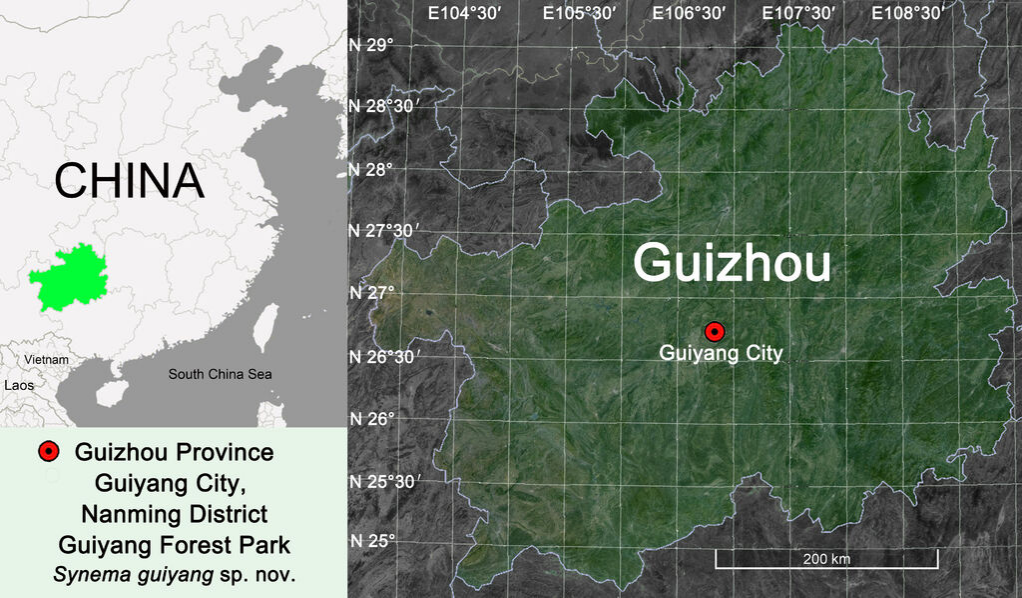
Distribution record of *Synemaguiyang* sp. nov. (red circle).

While examining spiders collected from Guiyang City, Guizhou Province, south-western China (Fig. 1), we found pairs of *Synema* specimens of crab spiders in the same location, which are with similar habitus, markings, leg spination and other characters (Fig. 3E, F and Fig. 4). Therefore, it is very likely they are opposite sexes of the same species. Subsequently, we matched the female and male together, based on DNA barcoding data. Additionally, we found that these specimens belong to an undescribed species similar to the Japanese species *S.albomaculatum* Ono, 2001 and *S.chikunii* Ono, 1983. Therefore, they are considered as new to science and are described under the name of *S.guiyang* sp. nov. The aim of the current paper is to describe the new species, providing detailed morphological descriptions and illustrations.

## Materials and methods

Specimens in this study were collected by hand collecting from leaf-litter in Guiyang Forest Park, Guiyang, Guizhou. Spiders were fixed and preserved in 95% ethanol. Specimens were examined with an Olympus SZX7 stereomicroscope; details were studied with an Olympus CX41 compound microscope. Female epigynes and male palps were examined and illustrated after being dissected. Epigynes were removed and cleared in warm lactic acid before illustration. The vulva was also imaged after being embedded in Arabic gum. Photos were made with a Cannon EOS70D digital camera, mounted on an Olympus CX41 compound microscope. The digital images were taken and assembled using the Helifocus 6.80 software package. The distribution map was generated with Google Earth Pro 7.3.2 (Google Limited Liability Company).

A DNA barcode was also obtained for the species matching. A partial fragment of the mitochondrial cytochrome oxidase subunit I (CO1) gene was amplified and sequenced for 21 specimens, using the primers LCOI1490 (5’-GGTCAACAAATCATAAAGATATTG-3’) and HCOI2198 (5’-TAAACTTCAGGGTGACCAAAAAAT-3’). For additional information on extraction, amplification and sequencing procedures, see Wheeler et al. (2016).

All measurements were obtained using an Olympus SZX7 stereomicroscope and given in millimetres. Eye diameters were taken at the widest point. The total body length does not include the length of the chelicerae or spinnerets. Leg lengths are given as total length (femur, patella, tibia + metatarsus, tarsus). Most of the terminologies used in text and figure legends follow Tang and Li (2010b).

All specimens are deposited at the Museum of Guizhou Education University, Guiyang, Guizhou, China (MGEU, curator Hao Yu).

## Taxon treatments

### Synema guiyang

J. Zhang, Q. Lu & H. Yu sp. n.

BB6AFCEC-3FC5-5F68-A0A0-CFB80E085396

C659D2A5-4612-4DCA-9EA3-1A43DBB0A5DC

#### Materials

- **Type status:** Holotype. **Occurrence:** recordedBy: Hao Yu; individualID: YHTHO002; individualCount: 1; sex: male; lifeStage: adult; behavior: foraging; preparations: whole animal (ETOH); associatedSequences: GenBank: ON435709; **Taxon:** order: Araneae; family: Thomisidae; genus: Synema; specificEpithet: *guiyang*; scientificNameAuthorship: J. Zhang, Q. Lu & H. Yu,; **Location:** continent: Asia; country: China; countryCode: CHN; stateProvince: Guizhou; county: Guiyang City; locality: Guiyang Forest Park; decimalLatitude: 26.55540319; decimalLongitude: 106.75898910; **Identification:** identifiedBy: Hao Yu; dateIdentified: 2021-11; **Event:** samplingProtocol: by hand; samplingEffort: 10 km by foot; year: 2021; month: 8; day: 10; **Record Level:** institutionID: MGEU; basisOfRecord: PreservedSpecimen
- **Type status:** Paratype. **Occurrence:** recordedBy: Hao Yu; individualID: YHTHO003; individualCount: 1; sex: female; lifeStage: adult; behavior: foraging; preparations: whole animal (ETOH); associatedSequences: GenBank: ON435708; **Taxon:** order: Araneae; family: Thomisidae; genus: Synema; specificEpithet: *guiyang*; scientificNameAuthorship: J. Zhang, Q. Lu & H. Yu,; **Location:** continent: Asia; country: China; countryCode: CHN; stateProvince: Guizhou; county: Guiyang City; locality: Guiyang Forest Park; decimalLatitude: 26.55540319; decimalLongitude: 106.75898910; **Identification:** identifiedBy: Hao Yu; dateIdentified: 2021-11; **Event:** samplingProtocol: by hand; samplingEffort: 10 km by foot; year: 2021; month: 8; day: 10; **Record Level:** institutionID: MGEU; basisOfRecord: PreservedSpecimen

#### Description

**Male** (holotype) (Fig. 3E and Fig. 4A–C). Total length 3.68; carapace 1.69 long, 1.59 wide; abdomen 1.99 long, 1.33 wide.

Carapace (Fig. 3E and Fig. 4A, C) yellowish-brown, sides brown, a pair of coffee-coloured paramedian stripes starting from behind PME and PLE, almost reaching the posterior margin. In dorsal view, anterior eye row (AER) slightly recurved, posterior eye row (PER) distinctly recurved, PER nearly as wide as AER. Eye sizes and interdistances: anterior median eyes (AME) 0.08, anterior lateral eyes (ALE) 0.15, posterior median eyes (PME) 0.07, posterior lateral eyes (PLE) 0.11; distance between AMEs (AME–AME) 0.16, distance between AME and ALE (AME–ALE) 0.14, distance between PMEs (PME–PME) 0.20, distance between PME and PLE (PME–PLE) 0.34. Length of median ocular quadrangle (MOQ) 0.37, MOQ anterior width 0.31, MOQ posterior width 0.32. Clypeal height 0.18. Chelicerae yellowish-brown, both margins with one tooth. Labium and endites coloured as chelicerae, endites depressed posteriorly, slightly convergent anteriorly, with dense scopulae on anterior margin; labium nearly hexagona, anterior margin with sparse setae. Sternum yellow, more or less cordiform, 0.86 long, 0.77 wide.

Abdomen (Fig. 4A–C) elongate-oval in dorsal view, tapering posteriorly, almost flat in profile, shield-shaped. Dorsum centrally with several pairs of grey spots; lateral with five pairs of white spots and with fuzzy pattern represented by numerous horizontal stripes or blotches; venter yellow, without distinct pattern; spinnerets brown.

Legs basically yellowish-brown (Fig. 3E), all legs with inconspicuous dark brown annuli in the distal parts of femur, patella, tibia and entire metatarsus. Leg length: I 9.29 (2.59, 3.22, 2.37, 1.11), II 9.22 (2.64, 3.22, 2.27, 1.09), III 4.33 (1.33, 1.61, 0.88, 0.51), IV 4.88 (1.31, 1.70, 0.94, 0.53).

**Figure 2.**
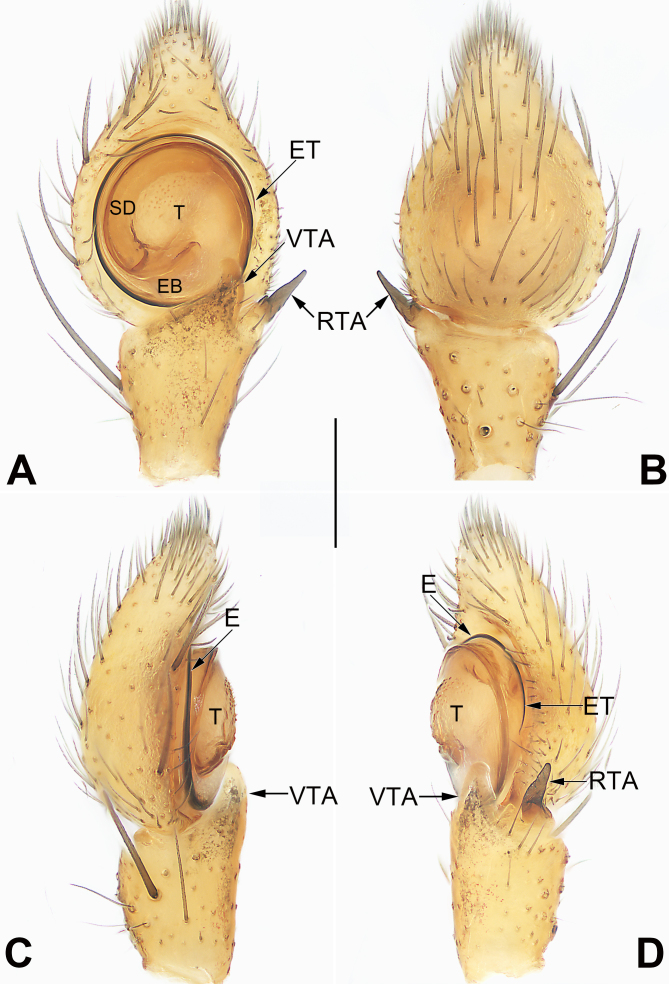
Male left palp of the holotype of *Synemaguiyang* sp. nov. **A** Ventral view; **B** Dorsal view; **C** Prolateral view; **D** Retrolateral view. Abbreviations: E = embolus; EB = embolar base; ET = embolar tip; ITA = intermediate tibial apophysis; RTA = retrolateral tibial apophysis; SD = sperm duct; T = tegulum; VTA = ventral tibial apophysis. Scale bar: 0.2 mm (equal for A–D).

Palp (Fig. 2A–D). Tibia relatively long, more than 1/2 of cymbium length, with three apophyses: a thick, thumb-shaped ventral one (VTA), ca. 1/3 of palpal tibia length; a distinctly small intermediate apophysis (ITA), triangular or dentiform in prolateral view, nearly invisible in ventral view, ~ 1/3 VTA length; and a relatively thin, spiny retrolateral apophysis (RTA). Tegulum circular and relatively flat, anterior part with numerous scale-like furcella; sperm duct (SD) distinct, forming a loop along tegular margin. Embolus (E) filiform, spiralled along tegular margin, but separated from tegulum; embolar base (EB) situated posterior margin of the tegulum (ca. 6 o’clock position), embolar tip (ET) terminated at the retrolateral flank (ca. 3 o’clock position).

**Figure 3.**
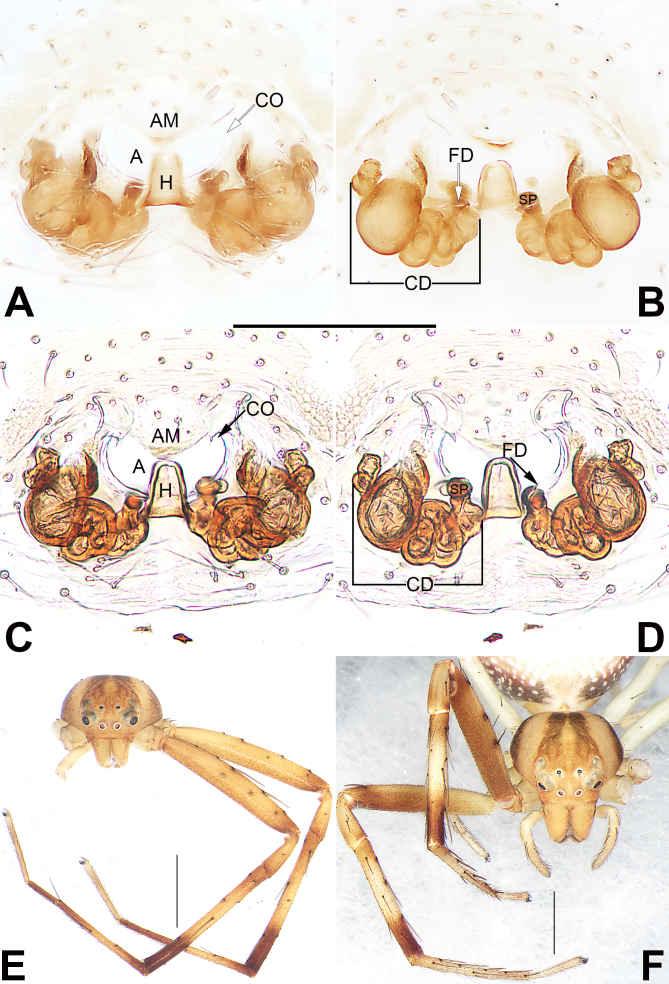
*Synemaguiyang* sp. nov., female paratype and male holotype, epigyne (**A–D**), frontal views of prosoma (**E**–**F**). **A**–**B** Macerated epigyne, ventral and dorsal; **C**–**D** Epigyne, macerated and embedded in Arabic gum, ventral and dorsal; **E** Male; **F** Female. Abbreviations: A = atrium; AM = atrial membrane; CD = copulatory duct; CO = copulatory opening; FD = fertilisation duct; H = hood; SP = spermatheca. Scale bars: 0.2 mm (equal for A–D); 1 mm (E, F).

**Figure 4.**
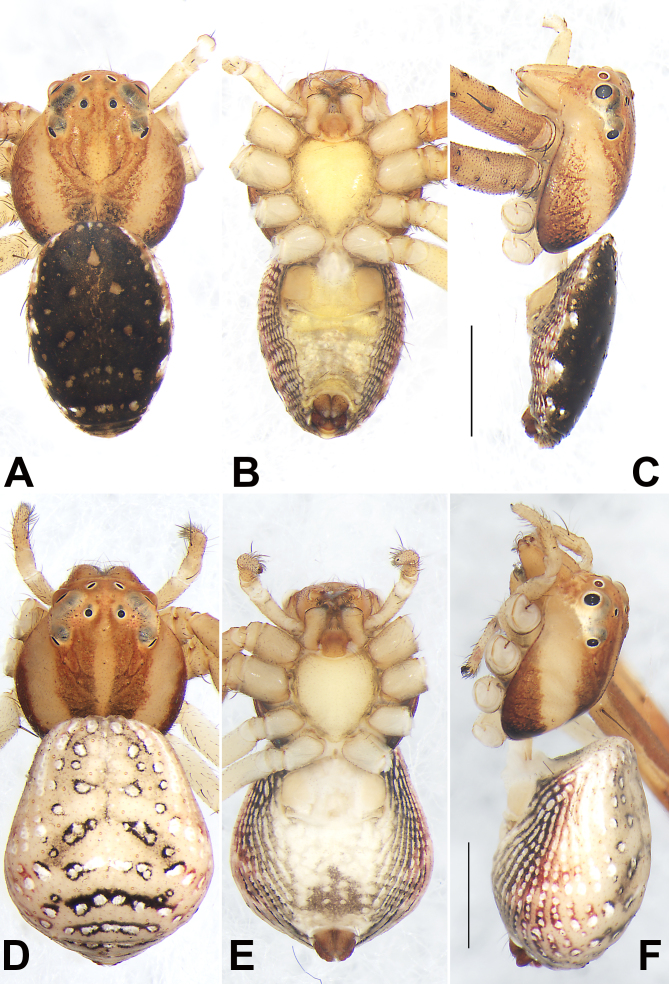
Habitus of *Synemaguiyang* sp. nov., male holotype (**A–C**) and female paratype (**D–F**). **A, D** Dorsal view; **B, E** Ventral view; **C, F** Lateral view. Scale bars: 1 mm (equal for A–C, equal for D–F).

**Female** (Fig. 3F and Fig. 4D–F). Total length 3.69; carapace 1.61 long, 1.53 wide; abdomen 2.08 long, 1.86 wide. Eye sizes and interdistances: AME 0.08, ALE 0.14, PME 0.06, PLE 0.10; AME–AME 0.18, AME–ALE 0.17, PME–PME 0.23, PME–PLE 0.36. MOQL 0.37, MOQA 0.32, MOQP 0.35. Sternum 0.79 long, 0.75 wide. Measurements of legs: I 6.04 (1.57, 1.77, 1.22, 0.74), II 7.50 (2.02, 2.50, 1.48, 0.75), III 3.45 (0.93, 1.09, 0.61, 0.41), IV — (1.04, 0.69, —, —). General characters as in male, but slightly larger in size and lighter in colour.

Epigyne (Fig. 3A–D). Epigynal plate distinctly wider than long, anterior and lateral margin not delimited, posterior margin rebordered; the arrangement of the various parts of the vulva are clearly visible through the tegument in ventral view. Epigynal plate with an atrium (A) and a hood (H). Atrium large and nearly crescent-shaped, anteriorly located, anterior margin with a triangular membrane (AM), posterior margin distinctly procurved. Hood bell-shaped, located at central portion of epigynal plate, its anterior margin overpasses the posterior margin of atrium. Copulatory openings (CO) indistinct, located antero-laterally to atrial borders, leading to parallel copulatory ducts (CD) directed posteriorly and then running transversely to connect with centrally located spermathecae (SP). Copulatory ducts sac-shaped, strongly twisted with several clearly visible constrictions, forming 3 ~ 4 balloon-shaped structures. Spermathecae distinctly small, papilliform or globular, located on the both sides of the hood, separated by about two diameters. Fertilisation ducts (FD) short and curved, acicular, located on dorsal surface of spermathecae.

**DNAbarcode**: 5'TATTTGGAGCTTGATCTGCTATAGTAGGGACAGCTATAAGAGTGTTAATTCGTATGGAATTAGGA AGATCTGGAAGATTATTAGGAAATGATCATCTTTATAATGTAATTGTTACCGCTCATGCTTTTGTTATGATTTTTTTTATA GTAATACCTATTTTAATTGGGGGTTTTGGAAATTGATTAGTACCTTTAATGTTAGGGGCTCCTGATATATCTTTCCCTCG GATGAATAATTTATCTTTTTGATTATTACCCCCTTCATTATTTTTATTATTTATATCTTCTATAGTAGAGGTAGGTGTAGGG GCAGGATGAACTGTTTATCCTCCTTTAGCTTCTAGAGTTGGGCATATAGGAGGATCTATAGATTTTGCTATTTTTTCTTT ACATTTAGCTGGAGCTTCTTCTATTATAGGAGCGGTTAATTTTATTTCTACTATTATTAATATACGAACTAGAGGTATAAG AATAGAAAAGGTTCCTTTGTTTGTATGATCTGTATTAATTACAGCTATTTTACTTCTTTTGTCTTTACCTGTATTAGCAGG TGCTATTACTATATTATTAACTGATCGTAATTTTAACACTTCTTTTTTTGATCCTGCAGGGGGAGGGGATCCAATTTTATT TCAACATTTGTTTTGATTTTT3' (holotype, YHTHO002; GenBank: ON435709).

**DNAbarcode**: 5'TATTTGGAGCTTGATCTGCTATAGTAGGGACGGCTATAAGAGTGTTAATTCGTATGGAATTAGGA AGATCTGGAAGATTATTAGGAAATGATCATCTTTATAATGTAATTGTTACCGCTCATGCTTTTGTCATGATTTTTTTTATA GTAATACCTATTTTAATTGGGGGTTTTGGAAATTGATTAGTACCTTTAATGTTAGGGGCTCCTGATATATCTTTCCCTCG GATAAATAATTTATCTTTTTGATTATTACCCCCTTCATTATTTTTACTATTTATATCTTCTATAGTAGAGGTAGGTGTGGGG GCAGGATGAACTGTTTATCCTCCTCTAGCTTCTAGAGTTGGGCATATAGGAGGATCTATAGATTTTGCTATTTTTTCTTT ACATTTAGCTGGGGCTTCTTCTATTATAGGGGCGGTTAATTTTATTTCTACTATTATTAATATACGAACTAGAGGTATAAG AATAGAAAAGGTTCCTTTGTTTGTATGATCTGTATTAATTACAGCTATTTTACTTCTTTTGTCTTTACCTGTATTAGCAGG TGCTATTACTATATTATTAACTGATCGTAATTTTAACACTTCTTTTTTTGATCCTGCAGGGGGAGGGGATCCAATTTTATT TCAACATTTGTTTTGATTTTT3' (paratype, YHTHO003; GenBank: ON435708).

#### Diagnosis

The new species resembles *S.albomaculatum* and *S.chikunii* in having the similar habitus (abdomen dorsally with characteristic spots) (cf. Fig. 4A, D and Ono 2001: 230, figs. 65 and 69, Ono 1983: 60, figs. 1–3) and by the general shape of copulatory organs (filiform embolus spiralled along tegular margin, VTA thumb-shaped in males; copulatory duct sac-shaped with several clearly visible constrictions, spermathecae relatively small in females) (cf. Fig. 2A, D and Fig. 3B, D and Ono 2001: 230, figs. 66, 67, 71, Ono 1983: 60, figs. 4, 5, 7). *S.guiyang* sp. nov. can be distinguished from *S.albomaculatum* and *S.chikunii* by the following characters: for the males, embolus apically not curved, ITA distinctly smaller than RTA, apex of RTA relatively blunt and not bifurcate (Fig. 2A, B, D) (vs. embolus apically slightly curved; ITA absent, RTA apically sharp in *S.albomaculatum*, as in Ono 2001: 230, figs. 66–68; ITA slightly shorter than RTA, RTA apically bifurcate in *S.chikunii*, as in Ono 1983: 60, figs. 4, 5); for the females, epigyne plate with a large atrium, bell-shaped hood about 1/5 epigyne width (Fig. 3A, D) (vs. atrium absent, transverse band-shaped hood nearly as wide as epigynal plate, as in Ono 2001: 230, fig. 70 and Ono 1983: 60, fig. 6).

#### Etymology

The species name is derived from the name of the type locality; noun in apposition.

#### Distribution

Known from the Guiyang City, Guizhou Province, China (Fig. 1).

#### Biology

*Synemaguiyang* sp. nov. is a typical leaf-dwelling spider. The types were obtained from foliage in woods in the core zone of Guiyang Forest Park.

## Discussion

As two members of Talaini, the genus *Synema* is easily confused with siblings *Spilosynema* in general appearance. According to the diagnosis provided by Tang and Li (2010b), *Synema* and *Spilosynema* can be separated by some somatic characters: *Synema* judging from the globular abdomen dorsally covered with large dark brown markings and yellowish-white markings (vs. abdomen dorsally with characteristic white spots in *Spilosynema*); anterior metatarsi and tibiae with dense hairs (vs. few hairs in *Spilosynema*); PER slightly recurved (vs. distinctly recurved in *Spilosynema*). Strictly based on this diagnosis, it seems that the new species should be assigned to the genus *Spilosynema* (Fig. 3E, F and Fig. 4).

However, some genitalic characteristics are not mentioned in the diagnosis of Tang and Li (2010b): In *Spilosynema*, male palp with postero-prolateral cymbial apophysis and tutaculum, female epigyne with broad tube-shaped copulatory ducts (Tang and Li 2010b: figs. 49C, D, 50C, 51A, B, D, 52C, D, 53C, E, 54A, B, D, 55C, D, 56C, 57A, B, D, 58D, 59B). Actually, *S.guiyang* sp. nov. possesses no certain characters associated with the genus *Spilosynema*, due to lacking genitalic features just mentioned (Fig. 2A, B, D, Fig. 3B, D). In contrast, *S.guiyang* sp. nov. possesses several genitalic characters associated with the genus *Synema* and resembles some *Synema* species (including *S.albomaculatum*, *S.chikunii* and generotype, *S.globosum*; for a detailed diagnosis, see above). Therefore, it is very strange (or interesting) that *S.guiyang* sp. nov. exhibits typical somatic features of *Spilosynema* and genitalic features of *Synema*. In view of the fact that somatic characters are either poorly marked or variable, they are thus not sufficient for distinguishing the *Spilosynema* and *Synema*. Consequently, *S.guiyang* sp. nov. is assigned tentatively to the genus *Synema* in the present paper for the lack of a better solution.

## Supplementary Material

XML Treatment for Synema guiyang
